# Retinal Findings in Nonaccidental Abusive Head Trauma in an Infant: A Case Report

**DOI:** 10.7759/cureus.108702

**Published:** 2026-05-12

**Authors:** Nishanth S, Laxmi, Vishalakshi Kulkarni

**Affiliations:** 1 Department of Ophthalmology, Shri Dharmasthala Manjunatheshwara College of Medical Sciences and Hospital, Dharwad, IND

**Keywords:** abusive head trauma, battered baby syndrome, child abuse, fundoscopy, macular hole, nonaccidental injury, retinal hemorrhages, retinoschisis, roth spots, shaken baby syndrome

## Abstract

Nonaccidental head trauma, also known as abusive head trauma or battered baby syndrome, is a life-threatening form of child abuse. The ocular findings, particularly retinal hemorrhages (RH) and Roth spots, serve as critical diagnostic clues. Here, we report an extremely rare case of a nine-month-old male infant presenting with vomiting, seizures, and altered sensorium, in whom fundoscopic examination revealed innumerable Roth spots with multilayered RH extending to the periphery, bilateral concentric retinal folds with posterior hyaloid attachment suggestive of retinoschisis, and right eye subhyaloid hemorrhage with posterior hyaloid detachment. Follow-up revealed a full-thickness macular hole in the right eye. The CT scan showed bilateral subdural hemorrhage. Systemic investigations ruled out hematological, infectious, and cardiac etiologies. This cluster of findings was diagnostic of nonaccidental head trauma. This case underscores the indispensable role of ophthalmic examination in infants with unexplained neurological presentations and the need for a multidisciplinary approach in suspected child abuse.

## Introduction

Abusive head trauma (AHT), also known as shaken baby syndrome, is one of the most severe forms of nonaccidental injury inflicted upon infants and children. It encompasses a spectrum of intracranial injuries, including subdural hematoma, subarachnoid hemorrhage, and diffuse axonal injury combined with retinal hemorrhages (RH), resulting from violent acceleration-deceleration forces applied to the infant’s head [1,2]. RH are found in approximately 53%-80% of diagnosed cases of AHT [3]. This is a significant finding that goes in favor of AHT rather than an accidental head injury. This pattern of RH, particularly bilateral, multilayered, and extending to the periphery, has a strong diagnostic value [4]. Roth spots, which are RH with white fibrin-platelet centers, are relatively uncommon in isolated AHT, and their presence in this sort of presentation makes the case exceptionally rare [5]. We present a case of a nine-month-old infant with an uncommon ophthalmic presentation, characterized by numerous Roth spots, multilayered RH extending to the periphery, bilateral concentric retinal folds with retinoschisis, subhyaloid hemorrhage, and subsequent macular hole formation, in the setting of a suspected case of AHT.

## Case presentation

**Figure 1:**
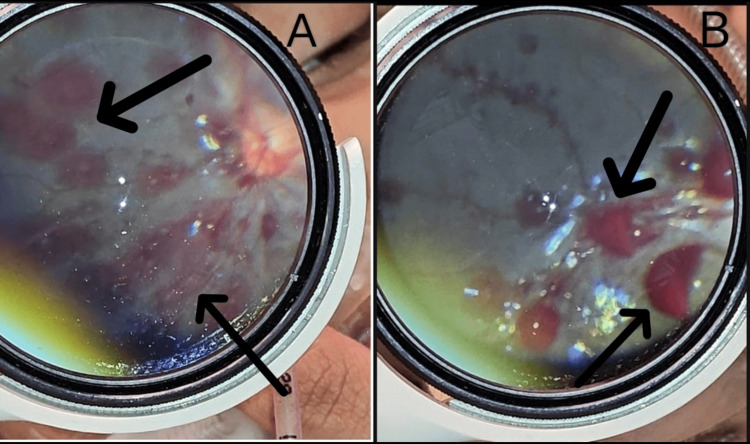
Numerous Roth spots with multilayered retinal hemorrhages extending to the periphery in both eyes (A) Right eye: multiple Roth spots and intraretinal blot hemorrhages noted. Peripapillary retinal hemorrhages are seen. (B) Left eye: multiple sub-ILM hemorrhages showing layering of the blood within the sub-ILM space ILM: internal limiting membrane

**Figure 2:**
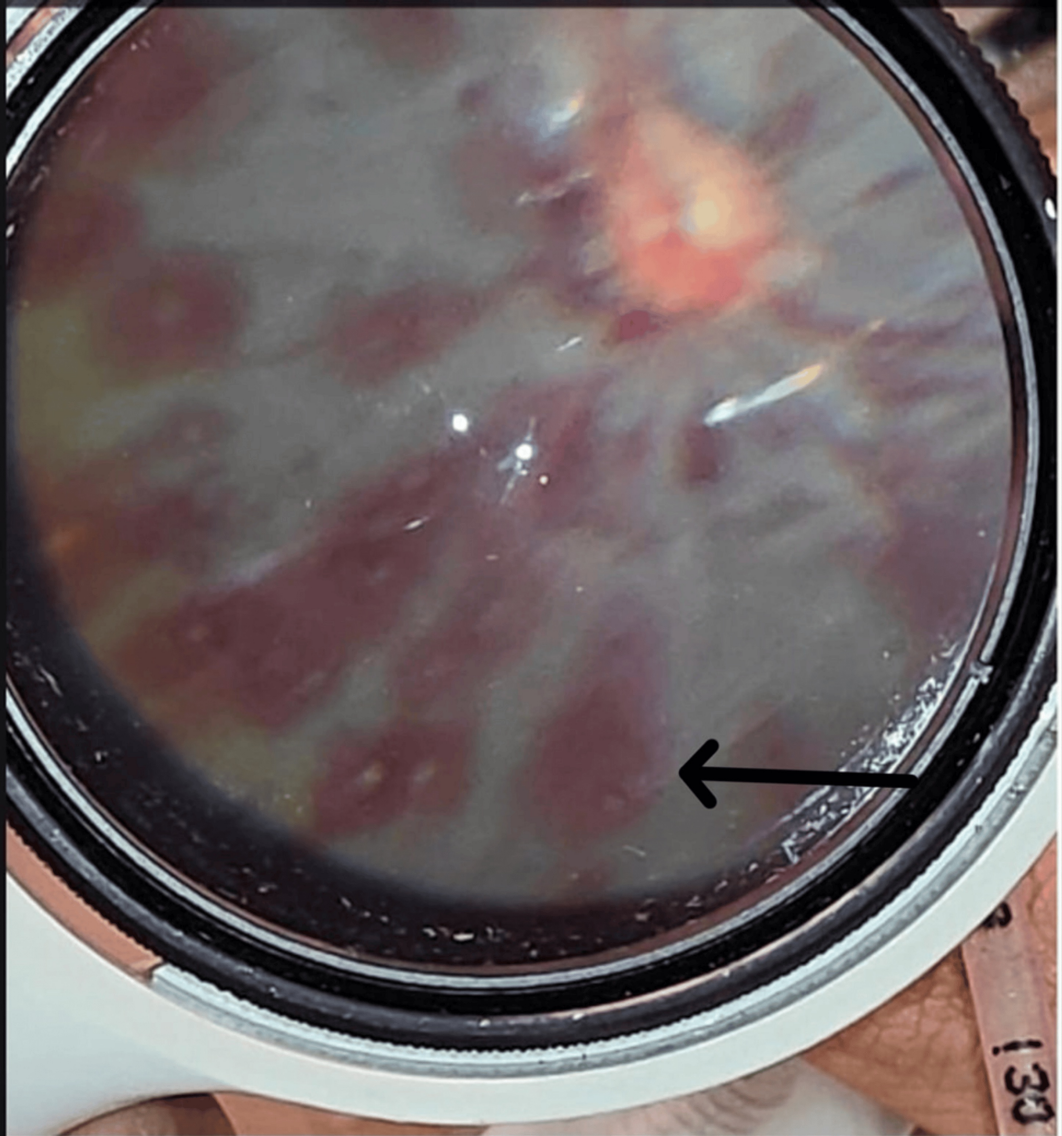
Concentric retinal folds with vitreomacular traction probably a retinoschisis

**Figure 3:**
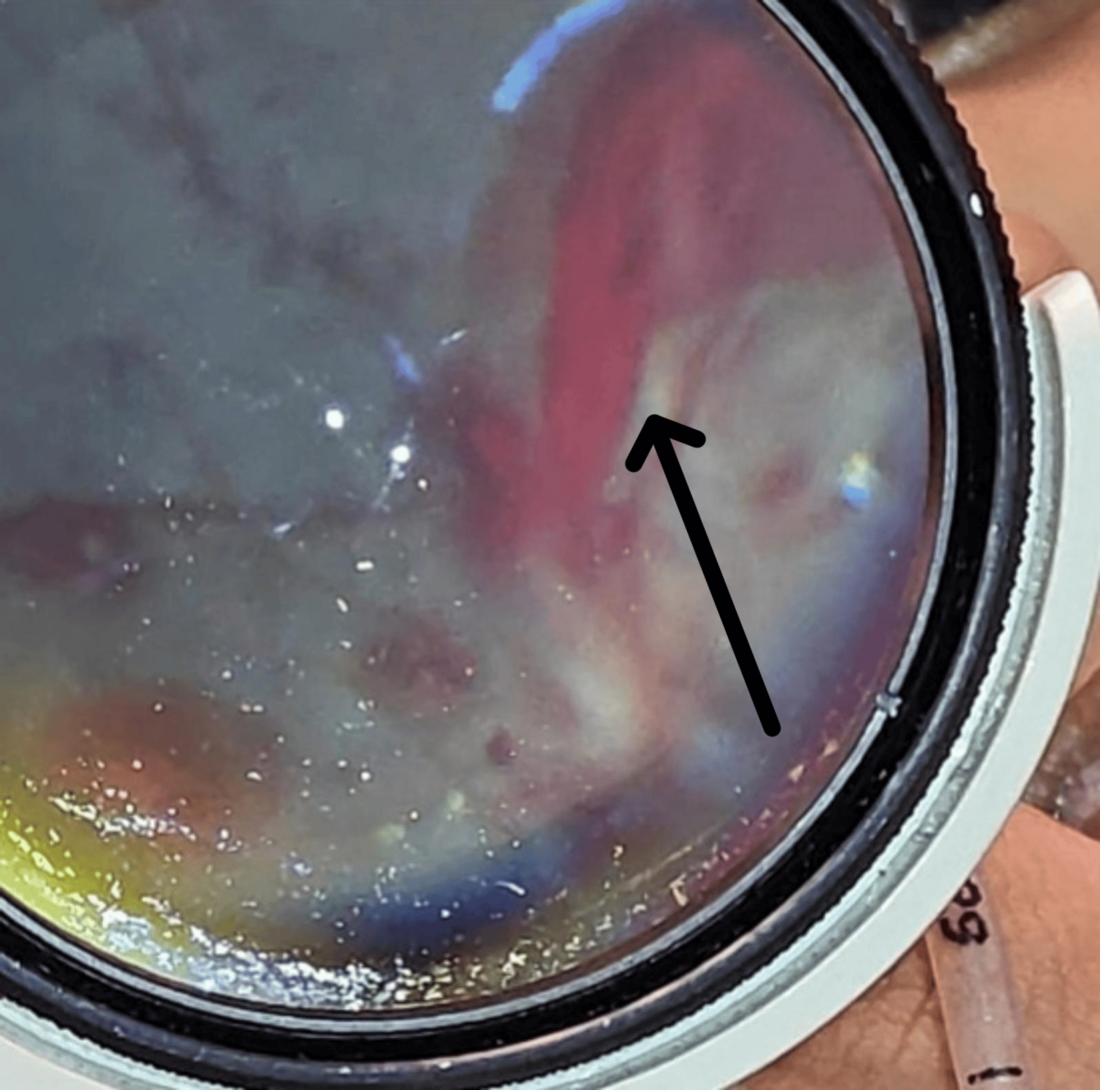
Right eye showing posterior hyaloid separation at the posterior pole with subhyaloid hemorrhage

A nine-month-old male infant was brought to the Emergency Department with complaints of one episode of vomiting, new-onset seizures, and a fixed staring look of one day's duration. There was no history of fever, trauma, or preceding illness as reported by the caregivers. The infant was the first child of nonconsanguineous parents with an uneventful birth and developmental history. On presentation, the infant was hemodynamically stable but showed signs of neurological compromise. He was intubated for airway protection. An ophthalmology consultation was sought to evaluate for raised intracranial pressure. Visual acuity could not be assessed as the patient was intubated. Anterior segment examination of both eyes was normal. Pupillary reaction was sluggish bilaterally. Fundoscopic examination was performed using indirect ophthalmoscopy under pharmacological mydriasis. A number of Roth spots (RH with white fibrin-platelet centers) along with multiple RH in different retinal layers, subretinal, intraretinal, and preretinal, extending from the posterior pole to the periphery, were seen in both eyes (Figure 1). Concentric retinal folds with vitreomacular traction were seen in both eyes (Figure 2). Posterior hyaloid separation at the posterior pole with subhyaloid hemorrhage was seen in the right eye (Figure 3).

**Figure 4:**
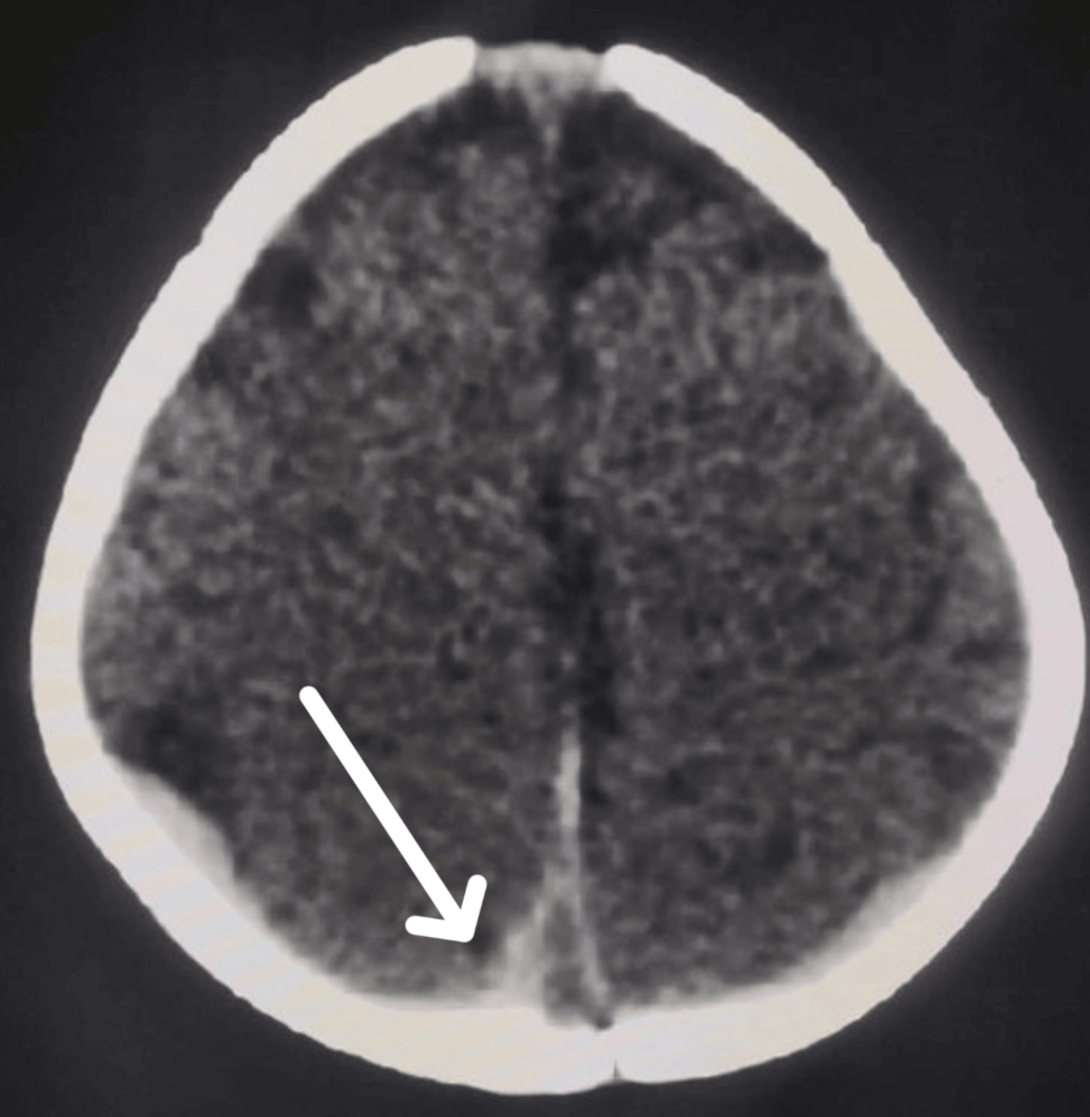
MDCT brain showing a thin rim of subdural hemorrhage along the bilateral parietal parafalcine and parieto-occipital regions MDCT: multidetector computed tomography

Systemic and laboratory investigations, including a comprehensive hematological evaluation with total leucocyte count, platelet count, and red blood cell count, were within normal limits. Peripheral blood smear showed normocytic normochromic anemia with lymphopenia. Activated partial thromboplastin time was mildly elevated but not clinically significant. Prothrombin time and international normalized ratio were normal. Bleeding disorders, including von Willebrand disease and hemophilia, were excluded. Echocardiography (2D Echo) was normal, ruling out cardiac causes of embolic Roth spots. Infectious workup, including TORCH titers, blood cultures, and viral serology, was negative. Brain imaging with multidetector computed tomography revealed a thin rim of subdural hemorrhage along the bilateral parietal parafalcine and parieto-occipital regions, without midline shift (Figure 4).

Repeat fundoscopy was performed 48 hours after the initial examination, and both eyes showed partial resolution of RH. However, the right eye demonstrated a new finding: an irregular full-thickness macular tear, likely due to traction secondary to the initially observed posterior hyaloid separation and subhyaloid hemorrhage. A multidisciplinary team comprising pediatric neurology, pediatric surgery, pediatric cardiology, and ophthalmology was involved. A full-body X-ray did not reveal any fractures. A detailed social history was obtained. There was an inconsistency in the history provided by caregivers regarding the mechanism of injury. After systematic exclusion of Valsalva retinopathy, including hematological (coagulopathy, thrombocytopenia), infectious (endocarditis, septicemia), metabolic (glutaric aciduria), and traumatic/accidental causes of bilateral RH, the combination of bilateral multilayered peripheral RH with Roth spots, traumatic retinoschisis, subhyaloid hemorrhage, macular tear, and bilateral subdural hemorrhages in an infant with inconsistent history was diagnostic of nonaccidental abusive head trauma (NAHT).

## Discussion

AHT is an underrecognized yet potentially lethal form of child abuse, predominantly affecting infants under two years of age [6]. The classic triad of AHT, subdural hemorrhage, encephalopathy, and RH, was largely fulfilled in our patient. Our case, however, is remarkable for the extraordinary extent and rarity of its ophthalmic findings [1].RH in AHT is thought to arise from sudden rises in intracranial and intraocular venous pressure, vitreoretinal traction, and Valsalva-type mechanisms during violent shaking [7]. The presence of hemorrhages extending to the periphery and in multiple retinal layers is strongly associated with AHT, as opposed to accidental trauma, where hemorrhages are typically few and confined to the posterior pole [4,8]. Roth spots, which are RH with white centers, are most classically described in bacterial endocarditis, leukemia, anemia, and septicemia [9]. Their occurrence in AHT is exceedingly rare and has been reported in only a few case reports. In our patient, cardiac (normal echo) and hematologic (normal counts, no coagulopathy) causes were conclusively excluded, making the Roth spots attributable to the traumatic event itself, possibly through local ischemia and fibrin-platelet deposition within hemorrhagic foci [5]. Traumatic retinoschisis is the splitting of the retinal layers and is considered almost pathognomonic of AHT when found in infants [10]. It results from rapid acceleration-deceleration forces causing vitreoretinal traction at the peripheral retina. Bilateral concentric retinal folds with posterior hyaloid attachment, as noted in our patient, represent a severe manifestation of this vitreoretinal tractional phenomenon.The development of a full-thickness macular tear as a sequela of subhyaloid hemorrhage and posterior vitreous detachment is an uncommon but recognized complication with significant implications for long-term visual prognosis. This case reinforces the critical principle that the ophthalmologist is often the first physician to uncover evidence of child abuse. Fundoscopy in an infant with unexplained neurological symptoms is not merely supplementary, but is diagnostic. The clinician must be systematic in excluding alternative diagnoses and must involve child protective services when AHT is suspected, as mandated by law in most jurisdictions [11].

The significance of this case is further amplified by its rarity in the South Indian population. Published literature on AHT from India, particularly from Karnataka and the broader South Indian region, remains extremely scant [12]. Cultural and societal barriers often result in underreporting or misclassification of nonaccidental injuries, making robust documentation of such cases critically important. The severity of ophthalmic involvement seen here, with innumerable Roth spots, multilayered peripheral RH, traumatic retinoschisis, subhyaloid hemorrhage, and macular hole formation occurring simultaneously in a nine-month-old infant, is, to our knowledge, among the most extensive presentations reported from this region. This case serves as an important sentinel event, highlighting the urgent need for heightened clinical awareness, systematic ophthalmic screening protocols, and stronger child protection frameworks within South Indian tertiary care centers [12,13].

## Conclusions

We present a relatively rare case of AHT in a nine-month-old infant manifesting as innumerable bilateral Roth spots with multilayered RH, traumatic retinoschisis, subhyaloid hemorrhage, and subsequent macular hole formation. The combination of these ophthalmic findings with neuroimaging evidence of bilateral subdural hemorrhages, in the absence of hematologic, infectious, or cardiac causes, established the diagnosis of NAHT. Early and systematic ophthalmic examination is indispensable in infants with unexplained neurological presentations. Ophthalmologists must maintain a high index of suspicion for AHT and work within a multidisciplinary framework to ensure prompt diagnosis, child protection, and optimal management.
